# The effect of KUS121, a novel VCP modulator, against ischemic injury in random pattern flaps

**DOI:** 10.1371/journal.pone.0299882

**Published:** 2024-12-26

**Authors:** Koichi Yoshimoto, Ryosuke Ikeguchi, Takashi Noguchi, Maki Ando, Daichi Sakamoto, Terunobu Iwai, Kohei Nishitani, Hanako Ohashi Ikeda, Akira Kakizuka, Shuichi Matsuda

**Affiliations:** 1 Department of Orthopaedic Surgery, Graduate School of Medicine, Kyoto University, Kyoto, Japan; 2 Department of Ophthalmology and Visual Sciences, Graduate School of Medicine, Kyoto University, Kyoto, Japan; 3 Laboratory of Functional Biology, Graduate School of Biostudies, Kyoto University, Kyoto, Japan; City of Hope National Medical Center, UNITED STATES OF AMERICA

## Abstract

Surgery using skin flaps is essential for soft tissue reconstruction. However, postoperative ischemic injury of the skin flap is a major complication and a top concern after the surgery. Currently, evidence-based drugs to fully prevent ischemic injury are not available. The purpose of this study was to evaluate the effect of KUS121, a VCP modulator, on flap ischemia using a rodent model. 26 Sprague-Dawley rats were randomly divided into two groups. The experimental group was intraperitoneally administered with 100 mg/kg KUS121 dissolved in 5% glucose solution 1 hour before surgery and once per day after surgery. The control group received the same amount of glucose solution on the same schedule. On day 7, 33.6 ± 3.7% of skin flaps in the control group had developed black necrosis compared with 26.4 ± 3.6% in the KUS121 group (p < 0.01). Immunohistochemistry showed that the KUS121 treatment reduced the number of apoptotic cells in the distal third of the flap (p < 0.01); moreover, in the KUS121-treated rats, the number of cells expressing CHOP, an endoplasmic reticulum (ER) stress marker, in the middle third of the flap was significantly lower than in the controls (p < 0.01). We examined the mRNA expression of *Ddit3* (CHOP) and *Casp3* (caspase-3) on day one after the surgery; mRNA expression of both genes appeared to decrease in the KUS121 group, as compared with the control group, although differences between groups were not significant. Thus, in a random pattern flap, KUS121 reduces ER stress and the number of apoptotic cells, thereby reducing ischemic damage of the flap.

## Introduction

Flap surgery is widely used to reconstruct wounds with skin defects. Lesions with extensive loss of skin or superficial soft tissue, such as those caused by trauma, must be covered with skin grafts or flaps. A major advantage of a skin flap over a free skin graft is that the skin flap has its own blood circulation and can survive without relying on a wound bed, whereas a free skin graft requires granulation tissue to form a wound bed [1]. A random skin flap is one type of skin flap; it does not have a specific vascular blood supply other than the subcutaneous vascular network. As a result, the distal blood supply is unstable, which often leads to localized necrosis [1,2]. Thus, the width-to-length ratio, which is the total length of the flap to the pedicle, is limited. In addition, even if an appropriate width-to-length ratio is preserved, patient factors such as vasoconstriction, diabetes, and smoking can cause flap necrosis [3,4], leading to infection and tissue defects. Patients with flap necrosis may require additional reconstructive surgeries to address these issues.

Various treatments have been tested to improve the viability of flaps [5–7]. For example, vasodilators such as nitroglycerin and sildenafil, sympatholytic agents such as phenoxybenzamine, and antithrombotics such as clopidogrel have been studied and their effects have been reported [8–11]. However, most of the treatments reported so far have been carried out in preclinical settings [7], and some of the drugs may have potential side effects. While an ideal tissue protection treatment, free from side effects, remains a desirable goal, to our knowledge, few have been clinically implemented to date.

Valosin-containing protein (VCP) is a hexameric protein belonging to the ATPases associated with diverse cellular activities (AAA) family. VCP is the most abundant soluble ATPase in mammalian cells, and therefore it is a major ATP consumer [12]. It is ubiquitously expressed in all types of cells and contributes, together with a variety of cofactors, to diverse intracellular activities. In the ubiquitin system, VCP segregates ubiquitinated proteins from their binding partners and cell membranes, leading to protein recycling and proteasome degradation. VCP is thought to act similarly on substances in the endoplasmic reticulum (ER) membrane and in chromatin. It also contributes to degradation related to mitochondria and lysosomes [13]. VCP mutations have been identified as causative for certain neurodegenerative diseases such as inclusion body myopathy associated with Paget disease of bone and frontotemporal dementia and rare cases of familial amyotrophic lateral sclerosis [14,15]. Moreover, pathogenic VCPs have been reported to exhibit significant elevation of ATPase activities compared with wild-type VCP [16]. These findings suggest that specific inhibitors of the ATPase activity of VCP may protect neuronal cells. After screening for novel ATPase inhibitors of VCP, a naphthalene derivative found to inhibit the ATPase activity of VCP without toxicity *in vitro* was discovered [17]. Kyoto University Substance (KUS) 121 was one of the compounds subsequently created by changing its chemical structure, and it clearly inhibited the ATPase activity of recombinant VCP *in vitro*. KUS121 specifically inhibits the ATPase activity of VCP without interfering with other cellular VCP functions [18]. KUS121 was reported to prevent cell death induced by ER stress [18]. To date, KUS121 has been reported to have a neuroprotective effect in murine glaucoma models, rat retinal ischemic injury models, and murine Parkinson’s disease models [19–21].

Since VCP is ubiquitous in cells, its application to myocardial ischemic models, renal ischemic models, and knee osteoarthritis models have revealed tissue-protective effects [22–24]. By extension, KUS121 could protect against other types of ischemic injuries, but its effects on skin flaps have not been evaluated. Furthermore, ER stress and apoptosis have been reported as important factors in ischemic injuries on skin flaps [7,25,26]. Based on these findings, a hypothesis was formulated suggesting that KUS121 may protect skin flaps against ischemic injuries. The purpose of this study was to evaluate the effect of KUS121 on random pattern flaps.

## Materials and methods

### Study design

Twenty-six Sprague-Dawley rats 10–12 weeks old (Charles River Japan, Kanagawa, Japan) were randomly divided into two groups: a KUS121-administered group and a control group. Each group contained 13 animals. In each group of 13, 6 rats were used for macroscopic and histological evaluations, 3 for immunohistochemical evaluations, and 4 for analysis of mRNA expression. Each rat was housed in an independent cage. All animals were housed at controlled room temperature (24°C) under a 14h light/ 10h dark cycle with ad libitum intake of food and water. All experiments were performed in accordance with the guidelines of the Animal Research Committee, Kyoto University Graduate School of Medicine (IACUC protocol number #MedKyo22247). Injections of the vehicle or KUS121 were administered by a blinded individual, distinct from the individual responsible for flap preparation. Additionally, macroscopic and histological evaluations were performed by separate blinded individuals, distinct from the one responsible for collecting macroscopic photographs and tissue samples.

### Creation of skin flaps

**Fig 1:**
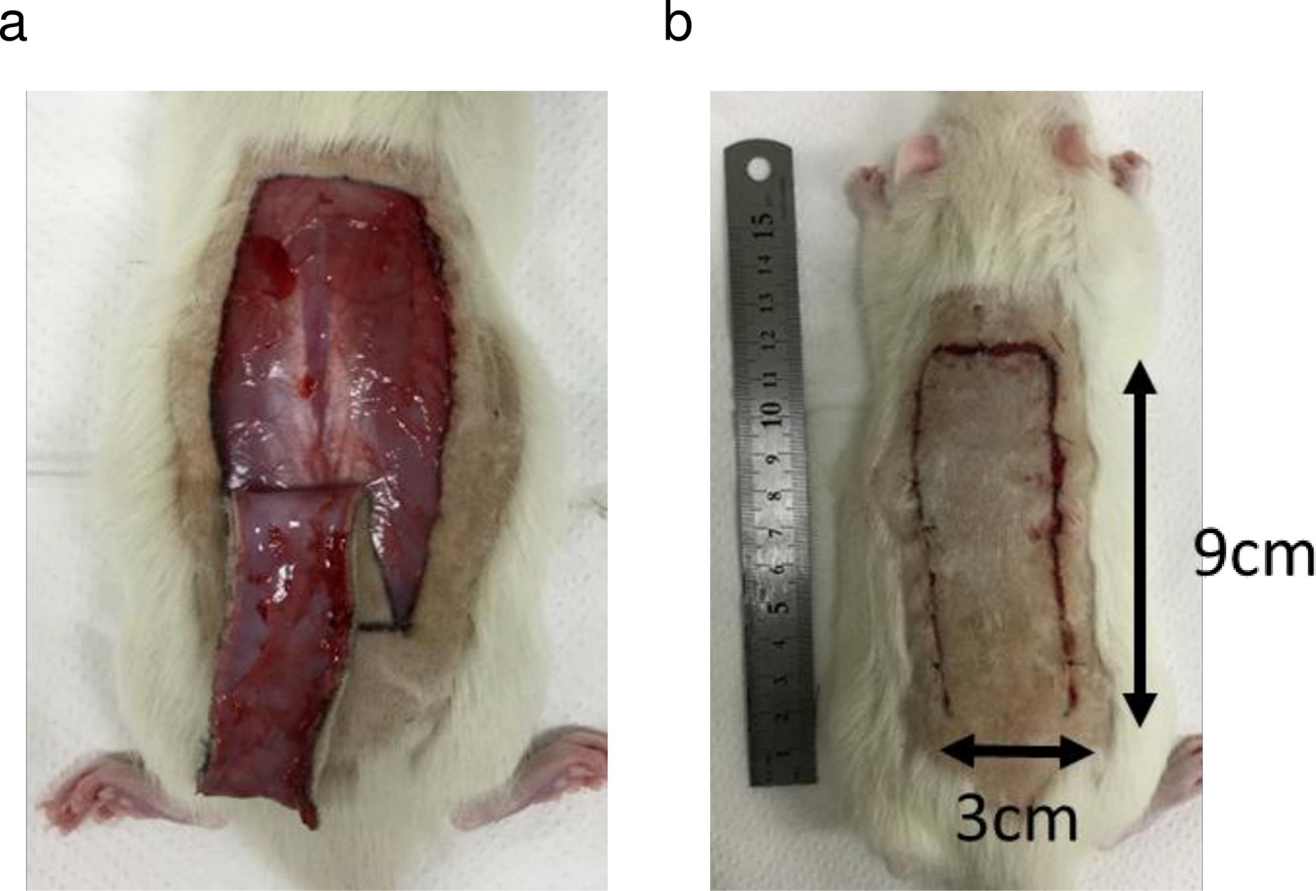
A random pattern flap was elevated on the dorsum of the rat along the longitudinal axis. The pedicle of the flap was placed on the caudal side. (a) The flap was elevated with the panniculus carnosus. (b) The elevated flap was sutured back to its original location.

Rats were anesthetized by administration of 4% isoflurane by inhalation with air for induction and maintenance with 2% isoflurane. After induction of anesthesia, carprofen 5 mg/kg was injected intraperitoneally for post-surgical pain relief. A heating pad was used and set at 40°C to maintain the body temperature of animals during surgery. The back of each rat was shaved to create random pattern flaps (McFarlane flaps), with the caudal side as the base of the skin flap. The base of the flap was set at the height of the iliac crest, the layer containing the skin and panniculus carnosus was peeled off, and a flap 3 cm wide and 9 cm long was elevated (Fig 1A). After elevation to the base, the flap was sutured back to its original site with a 4–0 nylon suture (Fig 1B).

### Drug administration

KUS121 was administered as a 10 mg/mL solution in 5% glucose. KUS121 was injected intraperitoneally, at a dose of 100 mg/kg, to rats in the KUS121-treated group 1 hour before flap elevation and once per day over the next 6 days after the surgery. The dose and times of administration of KUS121 is adjusted based on previous report [22]. In the control group, 5% glucose (vehicle) was injected intraperitoneally at 10 mL/kg, with the same schedule as for the KUS121-treated group.

### Macroscopic and histological evaluation

Six animals in each group were euthanized using high-concentration isoflurane on postoperative day 7. Photographs of the skin flaps were taken, and the injured and surviving areas were evaluated. The area that turned black was considered the injured area, and the ratio of the injured area to the entire skin flap area was calculated. Each area was calculated using ImageJ (National Institutes of Health, Bethesda, Maryland, United States).

Each flap was divided into thirds [27], from the proximal end of the flap to the distal end, designated Zones 1, 2, and 3, and the central skin of each zone was harvested. The harvested tissue was fixed in 4% paraformaldehyde at 4°C for 24 hours and then embedded in paraffin. The embedded tissue was sliced at a thickness of 5 μm and stained with hematoxylin and eosin. The stained slides were observed under an optical microscope (Eclipse 80i, Nikon, Tokyo, Japan) in three random fields of view at 200× magnification, and blood vessels were counted in each field of view to calculate blood vessel density.

### Immunohistochemical evaluation

Three animals in each group were sacrificed using high-concentration isoflurane on day 3 after surgery. The harvested tissues were fixed and embedded in paraffin.

The embedded tissue was sliced at a thickness of 5 μm and stained with TdT-mediated dUTP-biotin nick-end labeling (TUNEL). The TUNEL staining was performed using an In Situ Apoptosis Detection Kit (Takara Bio, Kusatsu, Japan) according to the manufacturer’s protocol. Slides were stained with ‘,3’-diaminobenzidine (DAB) substrate (Takara Bio) and counterstained with hematoxylin. Three random fields from each sample were observed with an optical microscope at 400× magnification. The number of positive-stained cells in the dermis per field of view was counted and the average calculated.

Immunostaining of CHOP was performed using a Vectastain Elite ABC kit (Vector Laboratories, Burlingame, California, United States) according to the manufacturer’s protocol. Antigen retrieval was performed with HistoVT One (Nacalai Tesque, Kyoto, Japan) at 65°C for 40 minutes, endogenous enzymes were blocked with 3% H_2_O_2_ for 10 minutes at room temperature, and secondary antibodies were blocked with 5% goat serum (Vector Laboratories) for 20 minutes. A rabbit monoclonal anti-DDIT3 antibody (ab179823; Abcam, Cambridge, United Kingdom) was diluted 400-fold as the primary antibody, and anti-rabbit IgG goat polyclonal antibody (BA-1000; Vector Laboratories) was diluted 200-fold as the secondary antibody. ImmPACT DAB (Vector Laboratories) was used for staining; slides were then immersed in 4-fold diluted hematoxylin for 10 seconds for counterstaining. Three random fields of view from each sample were observed at 400× magnification, and the number of positive cells in the dermis per field was counted, and then averaged.

### mRNA evaluation

Four rats in each group were used for analysis of mRNA expression in the skin flaps. Twenty-four hours after surgery, animals were sacrificed with high-concentration isoflurane, and the skin samples were harvested. The harvested tissues were immediately frozen in liquid nitrogen.

After homogenizing the tissue, RNA was extracted using TriPure Isolation Reagent (Roche, Basel, Switzerland) according to the manufacturer’s protocol. Reverse transcription was performed using ReverTraAce qPCR master mix (Toyobo, Osaka, Japan) according to the manufacturer’s protocol. SYBR Green real-time PCR (Toyobo) was performed using Step One Plus (Thermo Fisher Scientific, Waltham, Massachusetts, United States). Each sample was analyzed in triplicate, and the relative gene expression of *Casp3* (encoding Caspase-3), *Ddit3* (CHOP), *Vegfa* (VEGF-A), *Kdr* (VEGFR-2), *Egf* (EGF), and *Egfr* (EGFR) to *Actb* (encoding β-actin) was determined using the ΔΔCt method, where Ct is the cycle threshold. Primer sequences are shown in Table 1.

**Table 1:** Primer sequences.

Gene	Primer sequence
*Actb* (β-actin)	Forward: 5′-CCCGCGAGTACAACCTTCT-3′
	Reverse: 5′-CGTCATCCATGGCGAACT-3′
*Casp3* (Caspase-3)	Forward: 5′-AATTCAAGGGACGGGTCATG-3′
	Reverse: 5′-GCTTGTGCGCGTACAGTTTC-3′
*Ddit3* (CHOP)	Forward: 5′-CCAGCAGAGGTCACAAGCAC-3′
	Reverse: 5′-CGCACTGACCACTCTGTTTC-3′
*Vegfa* (VEGF-A)	Forward: 5′-GAGTCTGTGCTCTGGGATTTG-3
	Reverse: 5′-TCCTGCTACCTCTTTCCTCTG-3′
*Kdr* (VEGFR-2)	Forward: 5′-TTACTGTCCAGCCTGCTAC-3
	Reverse: 5′-CCAAAGAGCGTCCAAGTTC-3′
*Egf* (EGF)	Forward: 5′-GGGAGGCTACAACTGC-3
	Reverse: 5′-GCAGCTTCCACCAACG-3′
*Egfr* (EGFR)	Forward: 5′-CGCTGGAGGAAAAGAAAGTT-3
	Reverse: 5′-GGATGGGGTTGTTGCTAAAT-3′

### Statistical analysis

The KUS121-treated group and the control group were compared and tested using Student’s *t*-test for macroscopic evaluation after confirming the equal variance by F-test. Welch’s *t*-test was employed for TUNNEL staining evaluation and CHOP immunostaining evaluation without hypothesis of equal variances. Mann-Whitney U-test was employed for vessel density evaluation and mRNA evaluation without hypothesis of the normal distribution. JMP Pro 16 (SAS Institute Inc., Cary, North Carolina, United States) was used for statistical analysis. The level of significance was set at p < 0.05.

## Results and discussion

To evaluate the effect of KUS121, we surgically created random pattern flaps on the dorsum of each rat, with the caudal side as the base of the skin flap (Fig 1). 100mg/kg KUS121 was administered intraperitoneally to the KUS121 experimental group 1 hour before flap creation and once per day on days 1 to 6 after surgery. In the control group, the same volumes of vehicle (5% glucose in distilled water) were administered in the same protocol.

**Fig 2:**
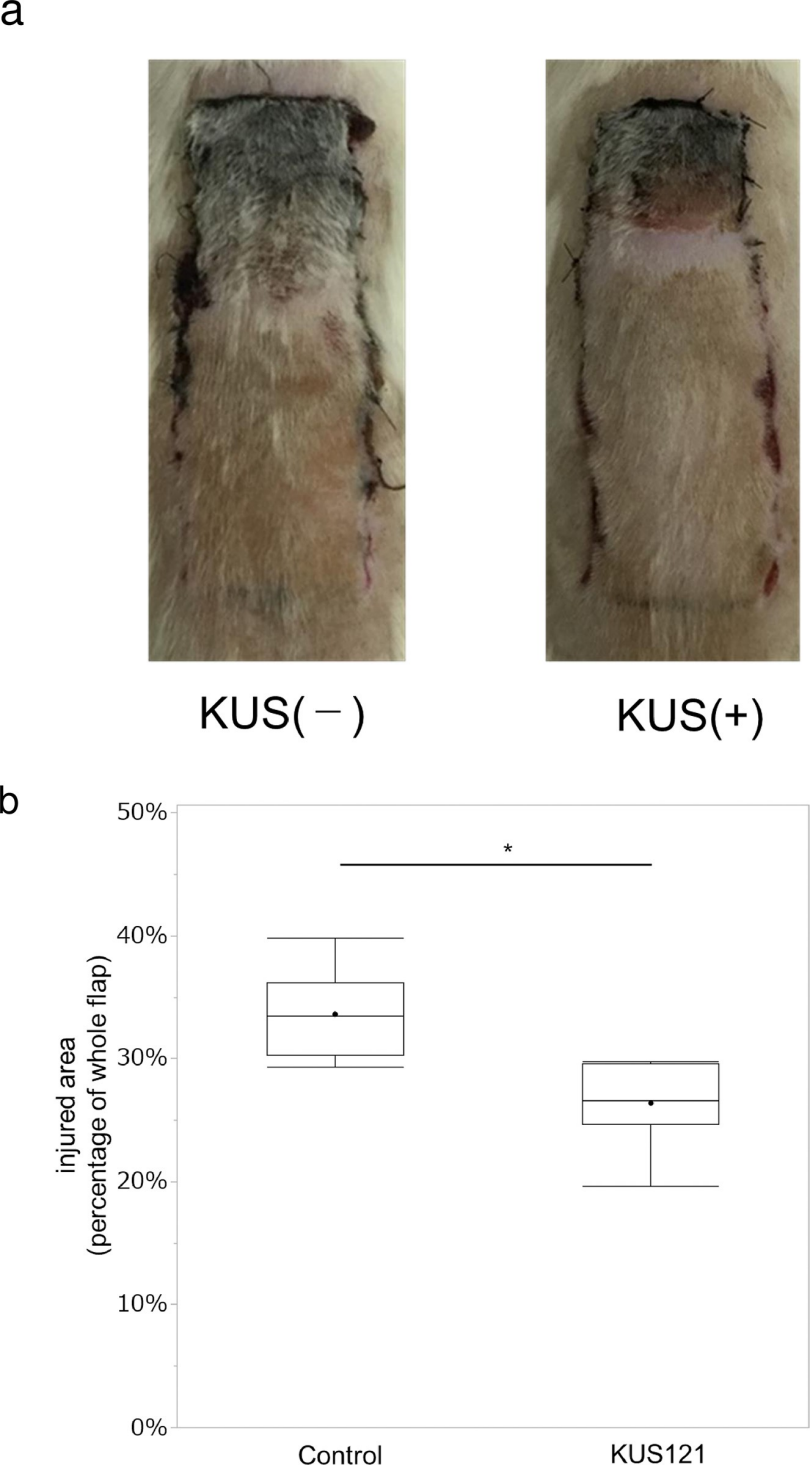
Flap damage on day 7. (a) Representative flaps from the control group and the KUS121-administered group. In each group, the distal side of the skin flap turned black. (b) The ratio of the injured area to whole flap area is shown. The boxes and lines inside each box indicate lower, upper quartile, and median; the whiskers indicate minimum and maximum value. The black dots indicate the mean. n = 6 for each group; * indicates a difference between groups (*p* < 0.01) by Student’s *t*-test.

In the macroscopic study, the distal end of the skin flaps turned black and became necrotic on day 7 after surgery. Fig 2A shows photographs of representative skin flaps in each group. The necrotic area was 33.6 ± 3.7% (mean ± standard deviation) in the control group and 26.4 ± 3.6% in the KUS121 group, indicating a significantly smaller necrotic area in the KUS121-treated group (p < 0.01; Fig 2B) (S1 Table.).

**Fig 3:**
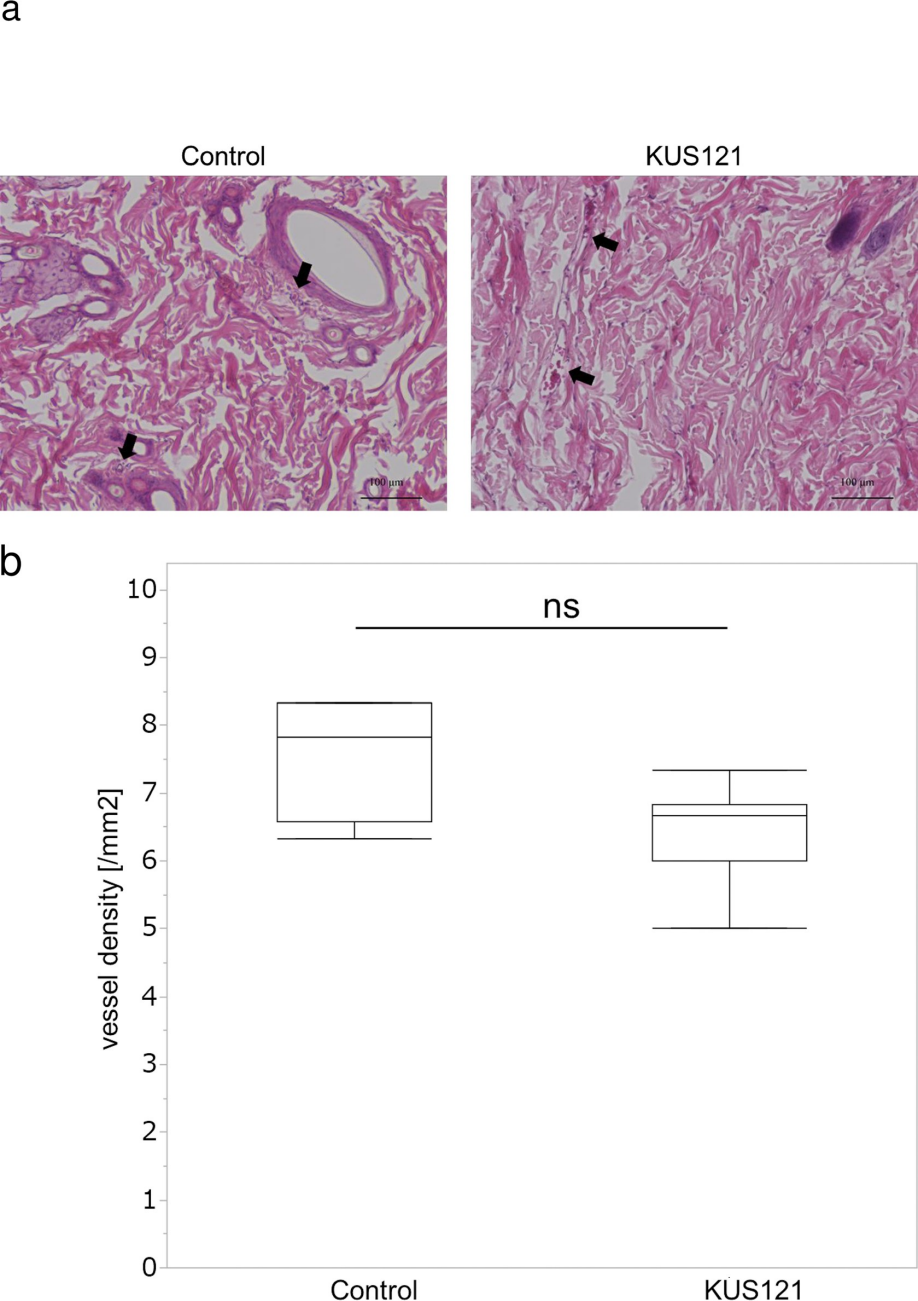
Skin tissues were collected on day 7 after surgery. (a) Arrows indicate blood vessels. (b) Mean blood vessel density was obtained by counting the number of blood vessels in each visual field observed at 200× magnification. Box and whisker plots indicate median, lower, and upper quartile, minimum and maximum value. Three random fields of view were observed for each tissue. n = 6 for each group. ns: Not significant (Mann–Whitney U-test).

Several agents reported to protect flaps against ischemia have been reported to have angiogenic effects [6,27,28]. On the other hand, as far as we know, there are no reports examining whether there are any angiogenic effects of KUS121, thus we investigated angiogenic effects of KUS121. Zone 1, the proximal area of the skin flap, showed the fewest visible changes, and tissue sections of Zone 1 were observed using a light microscope to determine whether there were any angiogenic effects of KUS121 administration (Fig 3A). The number of blood vessels in the dermis was counted in each field to calculate vessel density (Fig 3B) (S2 Table). The control group had a density of 7.6 ± 2.4/mm^2^, whereas the KUS121 group had a density of 6.4 ± 1.5/mm^2^, with no significant difference observed (p = 0.169) between the groups.

**Fig 4:**
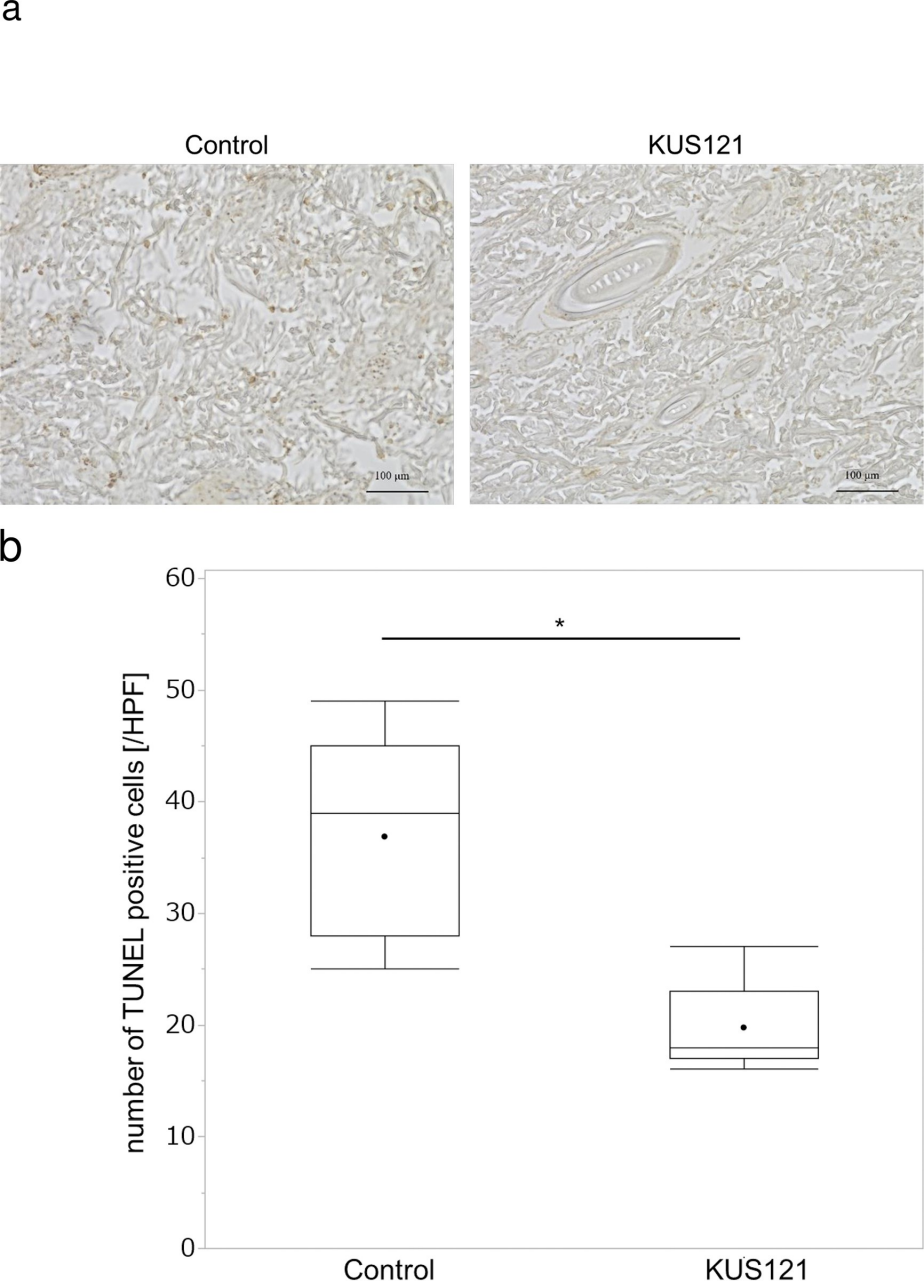
Skin tissues were collected on day 3 after surgery. (a) A representative section of a specimen from Zone 3 stained with TdT-mediated dUTP-biotin nick-end labeling (TUNEL) and observed at 400× magnification. Reddish brown cells are TUNEL-positive cells. (b) The number of TUNEL-positive cells in each visual field (high-power field, HPF) was counted. Box and whisker plots indicate median, lower, and upper quartile, minimum and maximum value. Three random fields of view were observed for each tissue. n = 3 for each group. *p < 0.01 (Welch’s *t*-test).

Next, we evaluated apoptotic cells present in the flap necrosis using immunohistochemical analysis. Because necrosis and apoptosis would be expected to be complete by day 7 after surgery, tissues for assessment of immunohistochemical analysis were harvested on day 3 after surgery [29]. Tissue sections were stained using TUNEL staining, and Zone 3, the distal area of the skin flap, was examined (Fig 4A). TUNEL-positive cells were found in the dermis and hypodermis, and those in the dermis were counted (Fig 4B) (S3 Table). The number of TUNEL-positive cells in the control group was 36.9 ± 8.8/high-power field (HPF) and that in the KUS121-treated group was 19.8 ± 3.7/HPF (p < 0.01).

**Fig 5:**
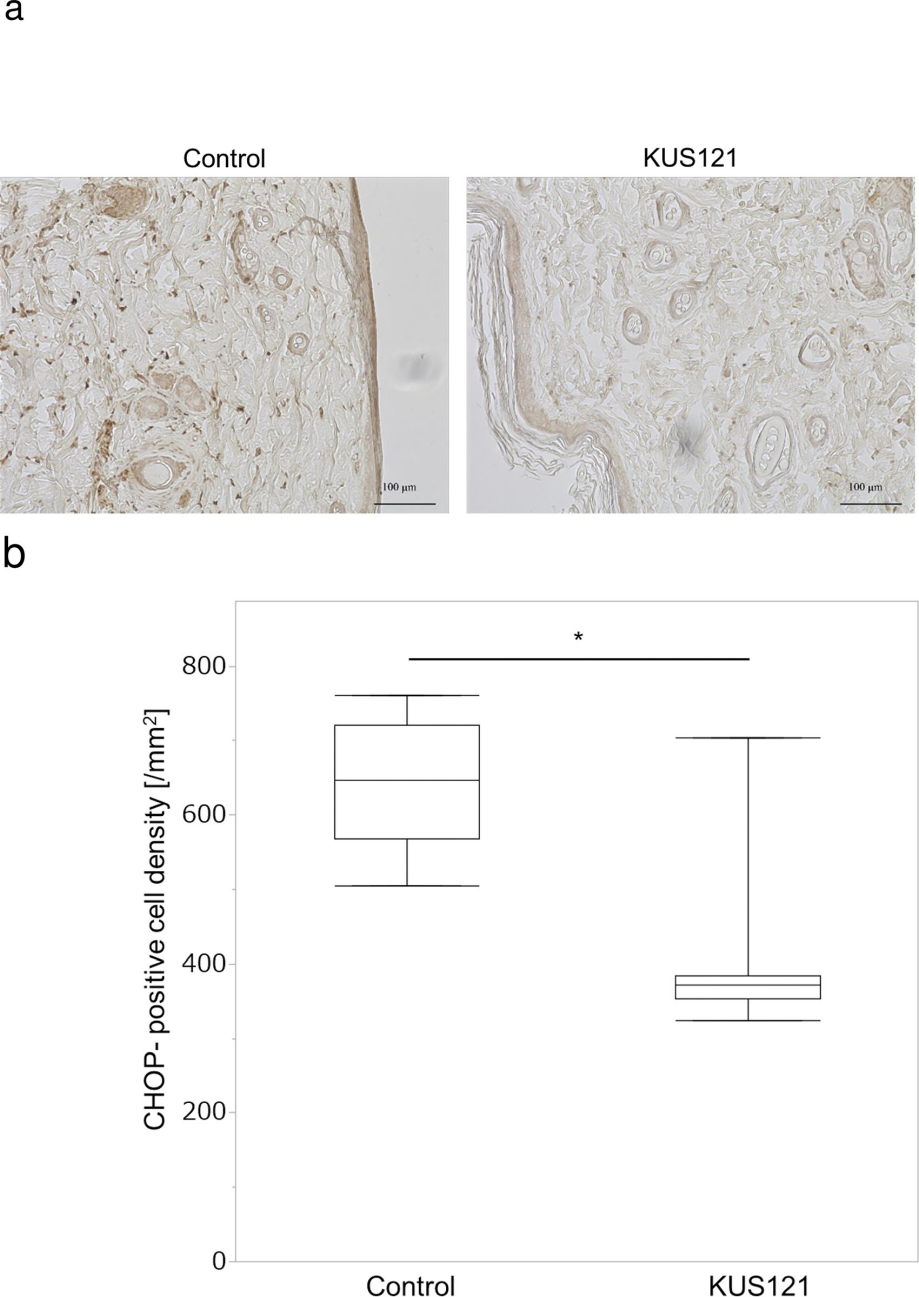
CHOP immunostaining of tissues harvested 3 days after surgery. (a) Representative sections from Zone 2 are shown in control and KUS121 groups. (b) CHOP-positive cell density in Zone 2. Box and whisker plots indicate median, lower, and upper quartile, minimum and maximum value. Three random fields of view were observed for each tissue. n = 3 for each group. *p < 0.01 (Welch’s t-test).

Similarly, tissue samples were immunostained to evaluate the expression of CHOP protein on day 3 after surgery (Fig 5A). CHOP-positive cells were found in the dermis and hypodermis, and CHOP-positive cells in the dermal layer were counted (Fig 5B) (S4 Table). In Zone 2, the middle area of the skin flap, the number of CHOP-positive cells in the control group was 646.8 ± 88.2/mm^2^ and that in the KUS121-treated group was 401.3 ± 114.9/mm^2^. The KUS121-treated group had significantly fewer CHOP-positive cells than the control group (p < 0.01).

**Fig 6:**
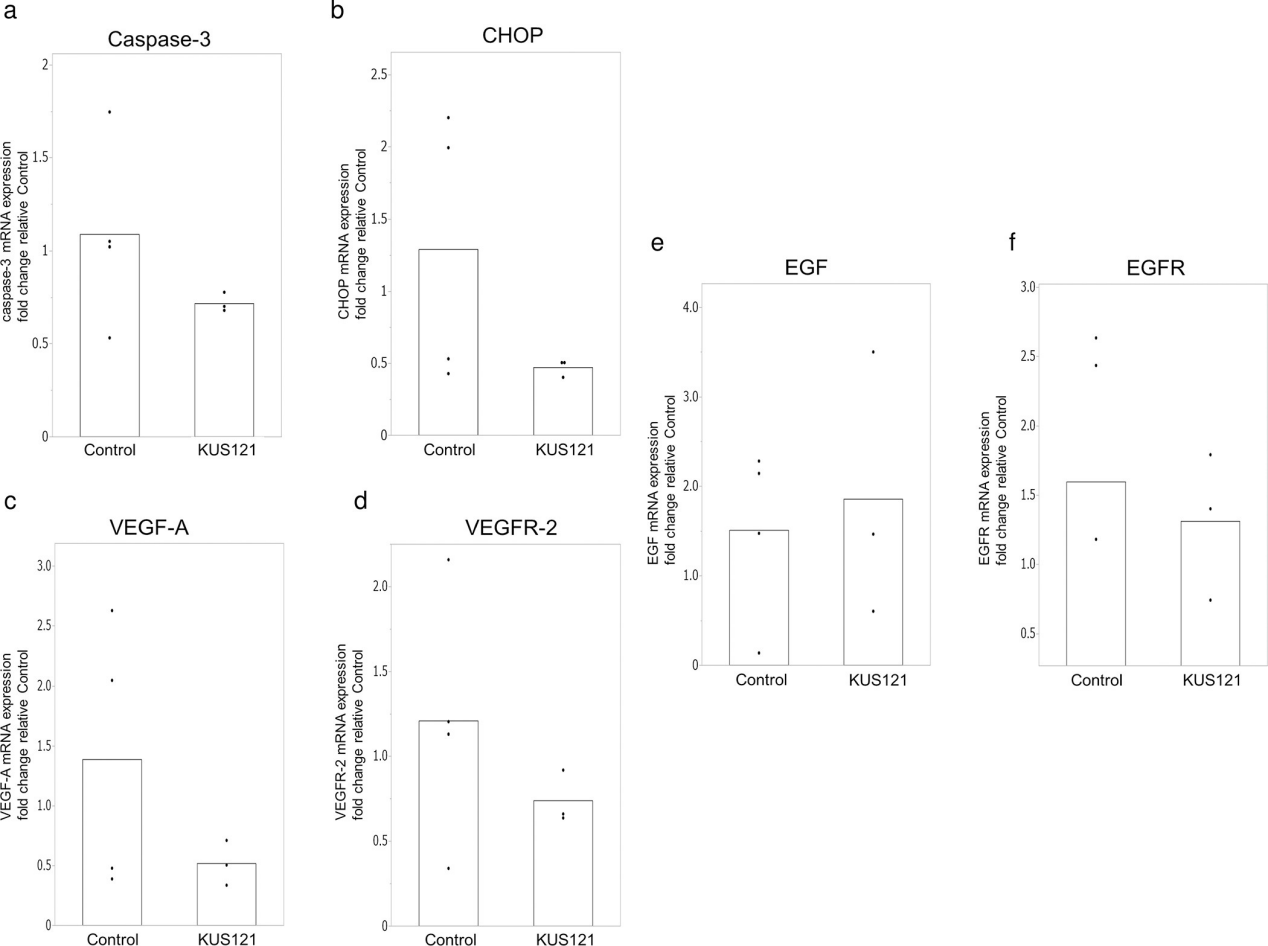
Expression of mRNA in the central flap 24 hours after surgery. (a) Expression of *Casp3* (encoding caspase-3). The bar indicates the mean of each biological replicates in two groups. Expression of each biological replicate were shown in scatter plots. (b) Expression of *Ddit3* (encoding CHOP) (c,d) Expression of *Vegfa* and *Kdr* (encoding VEGF-A and VEGFR-2). (e,f) Expression of *Egf* and *Egfr* (encoding EGF and EGFR). n = 4 for control group, and n = 3 for K.

We then evaluated the mRNA expression of *Casp3* (caspase-3), *Ddit3* (CHOP), *Vegfa* (VEGF-A), *Kdr* (VEGFR-2), *Egf* (EGF), and *Egfr* (EGFR). We assumed that mRNA expression in the tissues was likely to have reached a plateau on postoperative days 3 and 7; therefore, samples were examined 24 hours after surgery to assess earlier changes in gene expression. The experiment was conducted with 4 rats in the control group and 4 rats in the KUS121-treated group. In 24-hour postoperative specimens, the KUS121 group showed mean expression levels of *Casp3* and *Ddit3* that were 0.72-fold and 0.47-fold, respectively, relative to those in the control group (Fig 6) (S5 Table), with no significant difference found between groups. The KUS121 group also exhibited mean expression levels of *Vegfa*, *Kdr*, *Egf*, and *Egfr* that were 0.52-fold, 0.74-fold, 1.85-fold, and 1.31-fold, respectively, relative to that in the control group with no significant difference.

Flaps are classically divided into axial pattern flaps and random pattern flaps [30]. Axial pattern flaps include anatomical blood vessels along the skin flap, whereas random pattern flaps do not have significant blood vessel runs and are nourished instead by the subcutaneous vascular network. Random pattern flaps can be elevated without significant major vessels [1]. The length of the flap that can be safely elevated depends on the width of the pedicle; long flaps are associated with distal flap necrosis [31]. In this study, we used a rat dorsal flap (the McFarlane flap) as a model random pattern flap, which is widely used as an experimental model [32,33].

In the macroscopic study, rats administered KUS121 had a smaller necrotic area of the flap on day 7 after surgery compared with the control group. Suzuki et al. noted that a transient decrease in blood flow occurs within the random pattern flap immediately after surgery, and that blood flow gradually increases over the next 7 days [34]. KUS121 has been reported to preserve intracellular ATP levels against oxygen glucose deprivation in vitro [35] and reduce the area of necrosis in a cardiac ischemia-reperfusion model [8]. This protective effect against ischemia reperfusion injury after flap elevation may also occur in random pattern flaps.

Tissues on day 7 showed no significant difference in blood vessel density in the skin flap between the KUS121 group and the control group; these results establish that KUS121 had no significant effect on angiogenesis. Therefore, the flap-protective effect of KUS121 appears to be unrelated to angiogenesis.

In the immunohistochemical study, apoptotic cells were observed in the flap in both groups, but fewer apoptotic cells were observed in the KUS121-treated group. The presence of apoptosis in ischemic flaps was also reported by Fukunaga et al. [29]. They reported that oral administration of nitrosonifedipine reduces the number of apoptotic cells and increases the survival area in random pattern flaps via its antioxidative effects. The relevance of apoptosis in ischemia-reperfusion injury in the retina, kidney, and heart has been also noted, and KUS121-mediated pharmacological interventions have been reported to reduce apoptotic cells and organ injury [20,22,23,36–39]. Factors involved in flap necrosis include ischemia/reperfusion (I/R) injury, inadequate blood supply, and hemodynamic impairment. In addition, several stages are involved in the development of flap necrosis, including arterial constriction induced by vasoactive compound, loss of high energy phosphate molecules, formation of free radicals, deactivation of sodium and potassium pumps, endothelial edema, and further blockage of vessels by thrombosis [7]. Even in random pattern flaps, ischemia and subsequent reperfusion may occur immediately after flap elevation, and the flap may be protected by the suppression of apoptosis that occurs during this process. An additional clue came from our observation that the administration of KUS121 suppressed the expression of the C/EBP family protein CHOP in random pattern flaps. C/EBP family proteins are transcription factors that regulate many processes, including energy metabolism, immunity, inflammation, hematopoiesis, cell proliferation, and cell cycle and differentiation [40]. CHOP is thought to be involved in ER stress-induced apoptosis [41], and it has been suggested that ER stress and CHOP are also involved in ischemia-induced neuronal cell death [42]. Zhen et al. reported that in an ischemia-reperfusion model using an island flap in rats, administration of 4-phenylbutyrate decreased both the protein expression of CHOP and the number of apoptotic cells [25]. However, to our knowledge, no reports have shown a decrease in the necrotic area of the skin flap while reducing ER stress and apoptosis. Our results demonstrate that KUS121 can reduce skin flap necrosis through the reduction of apoptosis and ER stress.

In the evaluation of mRNA expression, while no significant differences were found between the KUS121 group and the control group, *Ddit3* (CHOP) and *Casp3* (caspase-3) mRNA levels tended to be lower in the KUS121-treated group than in the control group. Based on a previous report in which mRNA was evaluated in an ischemia reperfusion flap model [25], we evaluated the expression of mRNA 24 hours after surgery,. However, the flap model in this study was random pattern flap, thus the difference may not have been significant due to the timing of harvest or small number of samples. On the other hand, we evaluated the mRNA expression of growth factors such as vascular endothelial growth factor (VEGF) and epidermal growth factor (EGF) and their receptors. VEGF and EGF are important factors in flap angiogenesis and wound healing. Several treatments have been reported to protect the flap against ischemia and to promote wound healing while increasing these factors [43–45]. In our study, no significant differences were found in the mRNA evaluation of VEGF-A, VEGFR-2, EGF, and EGFR. These results are consistent with the finding that there was no difference in vascular density in the flaps on day 7, and may indicate that the effect of KUS121 is not related to angiogenesis.

This study has several limitations. First, we evaluated the effects of KUS121 administration up to day 7 but not beyond that timepoint. The timing of the evaluations and the size of flaps are not standardized across similar experiments [33]; however, we used the most common flap size and timings for our evaluations. The conditions affecting flap survival in clinical patients, such as the width-length ratio, may frequently be challenging, and patients may have additional factors like arteriosclerosis or metabolic disease. This study serves as a preliminary investigation to assess the effect on skin flaps under such conditions. Therefore, the most frequent flap size and healthy animal model were employed in this study [33]. KUS121 is found to have a protective effect for random skin flap in this study, further research in more severe conditions such as models with complications is expected. In addition, POD7 was employed as the endpoint for the macroscopic evaluation in this study. According to a review by Üstün et al., most experiments using random pattern flap models employed POD7 as the endpoint for flap survival [33]. The demarcation line of the injured area was clear on the 7th day in our experiment, which we thought was appropriate for evaluation. In addition, regarding immunohistochemical evaluation, one study reported the effect of nitrosonifedipine on skin flaps with evaluation of protein quantification and TUNEL staining, in which significant differences were observed on day 3 after surgery [29]. Yue et al. evaluated mRNA expression after administering 4-phenylbutyrate to axial pattern flaps over time up to 24 hours after surgery, and the greatest change was observed 24 hours after surgery [25]. This timepoint was adopted in our study. However, since the previous literature employed the axial pattern flap model, our random pattern flap model may exhibit different dynamics. To the best of our knowledge, however, there have been no reports on the dynamics of mRNA over time using random pattern flaps and there was no basis for evaluation at any other time. It is possible that the timing of this evaluation influenced the results of our mRNA analysis. In order to examine the appropriate timing for evaluation, it is necessary to evaluate the expression of mRNA over time for random pattern flaps, which was not done in our study. Second, the appropriate dosage and route of administration of KUS121 were not optimized in this study. Additionally, the side effects of KUS121 may not have been evaluated. KUS121 is a drug under development, and its pharmacokinetics are unknown. *In vitro* reports have stated that the cytoprotective effects of KUS121 against tunicamycin are observed in the range of 50–200 mM [18,22,24]. In this study, we adjusted the dosage, times, and route of administration of KUS121 based on these previous reports, however, more appropriate dosages and routes of administration likely exist. In the previous report, injection into the specific feeding artery was also performed, and its effectiveness was demonstrated [22]. Topical administration may reduce unknown KUS121 side effects. Especially, there is a possibility of administrating KUS121 via flap surface in contrast to internal organs. However, there are no reports on the possibility of topical administration or absorption through the skin. Additionally, local administration of the drug may cause physicochemical tissue damage due to the osmotic pressure and pressure caused by local injection. Therefore, systemic administration by intraperitoneal injection was selected in this study. However, although no animal death or injection site reactions were observed in our experiment, other systematic side effects could not be evaluated. To date, the clinical application of intravitreal injection to humans with central retinal artery occlusion has been reported [46], revealing potential side effects like iris neovascularization, macular edema, and worsening of retinal ischemia. However, systemic administration to humans has not yet been reported. Systemic evaluations, including blood analysis, are necessary when considering future clinical applications. Furthermore, while we conducted this preliminary study to explore the effect of KUS121 on skin flaps, it is necessary to investigate the basic pharmacokinetics of KUS121 as well.

Third, despite evaluating blood vessel density as an indicator of angiogenesis, we did not assess changes in blood flow after flap elevation. It has been reported that dynamic changes in blood flow occur immediately after flap elevation in random pattern flaps [34]. Dynamic blood flow changes in the flap can also be evaluated using laser Doppler [47,48]. Zhuang et al. used laser Doppler to observe dynamic changes after elevation in three skin flaps, including random pattern flaps, and found that the blood flow was recovered after a temporary decrease after surgery in the surviving area. They also observed irreversible blood flow decrease in the necrotic part of the skin flap [48]. The flap-protective effect of KUS121 may be based on the reduction of I/R injury, and even in random pattern flaps, KUS121 may be effective against transient blood flow reduction and subsequent injury in the border area of the viable area. These may be observed through dynamic blood flow evaluation in future studies.

Our current study does not elucidate the entire mechanism of KUS121 on skin flaps. Although we evaluated apoptosis and ER stress, these factors alone do not represent the complete mechanism of KUS121’s action on skin flaps. Furthermore, the extent to which these mechanisms contribute to macroscopic results remains unclear. Alternative methods, such as evaluating dynamic blood flow changes, may be necessary to uncover additional potential mechanisms in future research.

## Conclusions

KUS121 has been shown to preserve intracellular ATP, reduce apoptosis and protect retina, heart, kidney, and articular cartilage against ischemia [18,20,22–24,35,46,49,50]. Although numerous drugs have been tested for their effects on flap necrosis, none have been accepted as standard and used clinically. In conclusion, we have demonstrated that KUS121 has a protective effect against ischemia in random pattern flaps in the rodent model used in this study.

## Supporting information

S1 Table The raw data of Fig 2. The ratio of the injured area to whole flap area in each rat.
(DOCX)

S2 Table The raw data of Fig 3. The number of blood vessels in each visual field and their mean density.
(DOCX)

S3 Table The raw data of Fig 4. The number of TUNEL-positive cells in each visual field in each skin flap Zone 3.
(DOCX)

S4 Table The raw data of Fig 5. The CHOP-positive cell density [/mm2] in Zone 2 of each skin flap.
(DOCX)

S5 Table The raw data of Fig 6. The values of Ct and fold change of each mRNA in the central flap of each rat.
(DOCX)
