# Supplementary material for: The effect of KUS121, a novel VCP modulator, against ischemic injury in random pattern flaps
Source: PLoS One. 2024 Dec 26;19(12):e0299882. doi: 10.1371/journal.pone.0299882 (PMC11671021; doi:10.1371/journal.pone.0299882)
Supplement: S1 Table — The ratio of the injured area to whole flap area in each rat. (DOCX) [file pone.0299882.s001.docx]

Supporting Information

S1 Table. The raw data of Figure 2

| Group | injured area |
| --- | --- |
| control | 45.4% |
|  | 37.5% |
|  | 68.1% |
|  | 42.1% |
|  | 47.0% |
|  | 52.5% |
|  | 44.0% |
| KUS121 | 31.8% |
|  | 38.6% |
|  | 26.2% |
|  | 44.8% |
|  | 40.1% |
|  | 20.8% |
|  | 36.3% |
