# Supplementary material for: The effect of KUS121, a novel VCP modulator, against ischemic injury in random pattern flaps
Source: PLoS One. 2024 Dec 26;19(12):e0299882. doi: 10.1371/journal.pone.0299882 (PMC11671021; doi:10.1371/journal.pone.0299882)
Supplement: S2 Table — The number of blood vessels in each visual field and their mean density. (DOCX) [file pone.0299882.s002.docx]

Supporting Information

S2 Table. The raw data of Figure 3

| Group | field1 | field2 | field3 | mean |
| --- | --- | --- | --- | --- |
| control | 6 | 7 | 6 | 6.33 |
| control | 6 | 10 | 7 | 7.67 |
| control | 12 | 6 | 7 | 8.33 |
| control | 4 | 12 | 8 | 8.00 |
| control | 12 | 6 | 7 | 8.33 |
| control | 4 | 7 | 9 | 6.67 |
| KUS121 | 7 | 6 | 7 | 6.67 |
| KUS121 | 6 | 11 | 5 | 7.33 |
| KUS121 | 7 | 7 | 5 | 6.33 |
| KUS121 | 8 | 6 | 6 | 6.67 |
| KUS121 | 5 | 6 | 4 | 5.00 |
| KUS121 | 8 | 7 | 5 | 6.67 |
