# Supplementary material for: The effect of KUS121, a novel VCP modulator, against ischemic injury in random pattern flaps
Source: PLoS One. 2024 Dec 26;19(12):e0299882. doi: 10.1371/journal.pone.0299882 (PMC11671021; doi:10.1371/journal.pone.0299882)
Supplement: S3 Table — The number of TUNEL-positive cells in each visual field in each skin flap Zone 3. (DOCX) [file pone.0299882.s003.docx]

Supporting Information

S3 Table. The raw data of Figure 4

| Group | number of TUNELpositive cells [/HPF] |
| --- | --- |
| control | 30 |
|  | 39 |
|  | 26 |
|  | 43 |
|  | 33 |
|  | 25 |
|  | 40 |
|  | 49 |
|  | 47 |
| KUS121 | 23 |
|  | 16 |
|  | 18 |
|  | 27 |
|  | 16 |
|  | 18 |
|  | 18 |
|  | 19 |
|  | 23 |
