# Supplementary material for: The effect of KUS121, a novel VCP modulator, against ischemic injury in random pattern flaps
Source: PLoS One. 2024 Dec 26;19(12):e0299882. doi: 10.1371/journal.pone.0299882 (PMC11671021; doi:10.1371/journal.pone.0299882)
Supplement: S4 Table — The CHOP-positive cell density [/mm2] in Zone 2 of each skin flap. (DOCX) [file pone.0299882.s004.docx]

Supporting Information

S4 Table. The raw data of Figure 5

| Group | Number of CHOP positive cells [/mm2] |
| --- | --- |
| control | 624 |
|  | 728 |
|  | 504 |
|  | 708 |
|  | 589 |
|  | 545 |
|  | 713 |
|  | 646 |
|  | 760 |
| KUS121 | 351 |
|  | 324 |
|  | 380 |
|  | 379 |
|  | 358 |
|  | 386 |
|  | 703 |
|  | 356 |
|  | 372 |
