# Supplementary material for: The effect of KUS121, a novel VCP modulator, against ischemic injury in random pattern flaps
Source: PLoS One. 2024 Dec 26;19(12):e0299882. doi: 10.1371/journal.pone.0299882 (PMC11671021; doi:10.1371/journal.pone.0299882)
Supplement: S5 Table — The values of Ct and fold change of each mRNA in the central flap of each rat. (DOCX) [file pone.0299882.s005.docx]

Supporting Information

S5 Table. The raw data of Figure 6

|  | b-actin | Caspase-3 | Caspase-3 | CHOP | CHOP | VEGF-A | VEGF-A | VEGFR-2 | VEGFR-2 | EGF | EGF | EGFR | EGFR |
| --- | --- | --- | --- | --- | --- | --- | --- | --- | --- | --- | --- | --- | --- |
|  | Ct | Ct | fold change | Ct | fold change | Ct | fold change | Ct | fold change | Ct | fold change | Ct | fold change |
| control | 22.9933 | 28.2233 | 1.051 | 25.9300 | 1.995 | 23.1561 | 2.630 | 26.9936 | 1.131 | 32.1725 | 1.477 | 27.9067 | 2.634 |
| control | 20.7000 | 26.9100 | 0.533 | 25.8567 | 0.428 | 23.6213 | 0.389 | 26.4324 | 0.340 | 33.2944 | 0.138 | 29.9333 | 0.132 |
| control | 23.5600 | 28.8300 | 1.022 | 28.4067 | 0.531 | 26.1825 | 0.478 | 27.4702 | 1.204 | 32.2009 | 2.144 | 29.6300 | 1.182 |
| control | 22.8633 | 27.3600 | 1.747 | 25.6567 | 2.204 | 23.3880 | 2.046 | 25.9310 | 2.158 | 31.4144 | 2.282 | 27.8900 | 2.435 |
| KUS121 | 22.8767 | 28.7067 | 0.693 | 27.7867 | 0.508 | 24.9274 | 0.711 | 27.1752 | 0.920 | 30.8098 | 3.502 | 28.7000 | 1.402 |
| KUS121 | 21.5733 | 27.2367 | 0.778 | 26.8200 | 0.402 | 24.1172 | 0.505 | 26.3503 | 0.660 | 32.0397 | 0.605 | 28.3133 | 0.743 |
| KUS121 | 22.2633 | 28.1200 | 0.681 | 27.1933 | 0.501 | 25.3952 | 0.336 | 27.0902 | 0.638 | 31.4517 | 1.467 | 27.7333 | 1.791 |
